# A Performance/Cost Evaluation for a GPU-Based Drug Discovery Application on Volunteer Computing

**DOI:** 10.1155/2014/474219

**Published:** 2014-06-15

**Authors:** Ginés D. Guerrero, Baldomero Imbernón, Horacio Pérez-Sánchez, Francisco Sanz, José M. García, José M. Cecilia

**Affiliations:** ^1^National Laboratory for High Performance Computing, Center of Mathematical Modeling, University of Chile, 8370456 Santiago, Chile; ^2^Universidad Católica San Antonio de Murcia (UCAM), 30107 Guadalupe, Spain; ^3^Institute for Biocomputation and Physics of Complex Systems, 50018 Zaragoza, Spain; ^4^Computer Engineering Department, University of Murcia, 30100 Murcia, Spain

## Abstract

Bioinformatics is an interdisciplinary research field that develops tools for the analysis of large biological databases, and, thus, the use of high performance computing (HPC) platforms is mandatory for the generation of useful biological knowledge. The latest generation of graphics processing units (GPUs) has democratized the use of HPC as they push desktop computers to cluster-level performance. Many applications within this field have been developed to leverage these powerful and low-cost architectures. However, these applications still need to scale to larger GPU-based systems to enable remarkable advances in the fields of healthcare, drug discovery, genome research, etc. The inclusion of GPUs in HPC systems exacerbates power and temperature issues, increasing the total cost of ownership (TCO). This paper explores the benefits of volunteer computing to scale bioinformatics applications as an alternative to own large GPU-based local infrastructures. We use as a benchmark a GPU-based drug discovery application called BINDSURF that their computational requirements go beyond a single desktop machine. Volunteer computing is presented as a cheap and valid HPC system for those bioinformatics applications that need to process huge amounts of data and where the response time is not a critical factor.

## 1. Introduction

Integrating the latest breakthroughs in biochemistry, high performance computing, image processing, and computational modelling means enabling remarkable advances in the fields of healthcare, drug discovery, genome research, and so on. By integrating all these developments together, scientists are creating new exciting personal therapeutic strategies for living longer and healthier lifestyles that were unimaginable some short time ago.

The integration mentioned above spans many different areas, like life sciences, where there are many examples of scientific applications for discovering biological and medical unknown factors that could greatly benefit from increased computational resources. However, computing resources available on current systems are constrained, and thus this fact limits the the next step forward in this field. For instance, applications such as programs from the molecular modeling field used for visualizing molecular docking simulations and describing interatomic interactions for drug discovery such as* BINDSURF* [1] or protein folding applications that unlock the mystery of protein assembly and its relationship to cancers, Parkinson's disease, and Alzheimer's such as GROMACS [2] could clearly benefit from enhanced computing capabilities.

As can be seen, high performance computing technologies are at the forefront of those revolutions, making it possible to realize and accelerate radical biological and medical breakthroughs that would directly translate into real benefits for the society and the environment. In this regard, parallel computing technologies have brought dramatic changes into the high performance market [3]. Multicore CPUs (central processing unit) can now hold a dozen of cores, and many core GPUs (graphics processing unit) gather a myriad of stream processors. These components are being combined to build heterogeneous parallel computers offering a wide spectrum of high performance processing functions.

GPUs are massively parallel processors, which can support several thousand concurrent threads. Nowadays, many general-purpose applications from different fields have been successfully ported to these platforms achieving good speedups compared to their corresponding sequential versions [4–9]. The trend of using GPUs for general purpose computing has been favoured by the low cost of GPUs, mostly caused by the gaming business volume. GPUs are democratizing the high performance market, having a massively parallel chip for only $200.

Thus, large clusters are therefore adopting the use of these architectures, as a way to provide enough computational power to overcome the next century challenges [10]. However, current GPUs have a great impact on the power consumption of the system, as a high-end GPU may well increase the power consumption of a cluster node up to 30%, which is actually a big issue already. This is a critical issue especially for very large clusters, where the cost dedicated to power supply to such computers represents an important fraction of the total cost of ownership (TCO) [11, 12]. Besides, the carbon footprint from those supercomputers is on the rise, reaching the levels produced by the global airline industry, and experts estimate that pollutant emissions derived from the usage of these machines will quadruple by 2020 [13]. Virtualization techniques such as volunteer computing [14] may provide significant energy savings, as they enable a larger resource usage by sharing a given hardware among several users, thus reducing the required amount of instances of that particular device.

This paper evaluates a volunteer computing paradigm, based on the tuple* BOINC* [15] and Ibercivis, as an alternative to owning large GPU-based local infrastructures. We analyze several parameters such as performance, cost (including energy consumption, collocation cost, and machine market price), and availability of both architectures design. We illustrate this comparison for the execution of a representative GPU-based bioinformatics application called* BINDSURF* (we refer the reader to Ibercivis webpage (http://www.ibercivis.es/)).

The main contributions of this paper include the following.

- Although the elapsed time to obtain* BINDSURF* results in the volunteer computing platform is an order of magnitude slower than in our local infrastructure, the processing time is in the same range for both platforms thanks to the ubiquity of GPUs which are present in almost every desktop PC.
- The power consumption of our local infrastructure when executing BINDSURF is around 200 Watts. Moreover, additional costs for our local infrastructure need to be considered such as collocation cost and administration, while all of them are saved by the hardware donation of volunteers.
- The tuple* BOINC* and* Ibercivis* are presented as a valid alternative in the HPC arena for running bioinformatics applications, which are not real time but need many computational resources.

The rest of the paper is organized as follows.  Section 2 briefly introduces the preliminary knowledge necessary for the better understanding the experimental results in the rest of the paper. We also introduce the economic assumptions to assess our simulations. The experimental environment and the evaluation in both our local and volunteer computing infrastructure are shown in Section 3.  Section 4 shows the experimental results, offering an additional analysis of the cost of our local infrastructure, in terms of power consumption and economic impact, before we discuss these results in Section 5. Finally, in Section 6 we summarize our findings and conclude with suggestions for future work.

## 2. Materials and Methods

### 2.1. BINDSURF: High-Throughput Parallel Blind Virtual Screening

In this section, we introduce* BINDSURF* [1], an efficient and fast blind methodology for the determination of protein binding sites depending on the ligand that uses the massively parallel architecture of GPUs for fast prescreening of large ligand databases. We first briefly review the main characteristics of CUDA [16] for the benefit of readers who are unfamiliar with the programming model. CUDA is based on a hierarchy of abstraction layers; the thread is the basic execution unit; threads are grouped into blocks, each of which runs on a single multiprocessor, where they can share data on a small but extremely fast memory. A grid is composed of blocks, which are equally distributed and scheduled among all multiprocessors. The parallel sections of an application are executed as kernels in a SIMD (single instruction multiple data) fashion, that is, with all threads running the same code. A kernel is therefore executed by a grid of thread blocks, where threads run simultaneously grouped in batches called warps, which are the scheduling units.

*BINDSURF* divides the whole protein surface into arbitrary independent regions (also known as spots). Next, and thanks to the computational power provided by the efficient exploitation of the parallelism of GPUs, a large ligand database is screened against the target protein over its whole surface simultaneously, and docking simulations for each ligand are performed simultaneously in all the specified protein spots resulting in new spots found after the examination of the distribution of scoring function values over the entire protein surface. Using this approach, it has been found that* BINDSURF* predicts accurately and at an unprecedenteded speed the binding sites to which different ligands bind to the same protein in known cases that were problematic to other docking methods.

*BINDSURF* is a stochastic methodology that uses the Monte Carlo energy minimization scheme. One of the most important parameters is the number of Monte Carlo steps; very high values are preferred for this number so that we have more probabilities of finding the global minima of the potential energy surface and thus the accuracy of the prediction increases. Besides, high values for the number of Monte Carlo steps imply an increase in the number of required computations, so a compromise must be found for this number. We show later the use of different typical values for this parameter.

### 2.2. Volunteer Computing Paradigm Applied to Ibercivis

Volunteer computing (also called peer-to-peer computing or global computing) is a distributed computing approach where citizens offer their own computing resources to solve scientific projects. Recently, volunteer computing has moved to middleware systems that provide a distributed computing infrastructure independent of the scientific computation. Among them, the most popular one is BOINC, developed at the University of Berkeley. BOINC [15] provides to the scientific community the opportunity to use the computing power of thousands of CPUs and GPUs almost for free. BOINC provides a complete middleware system for volunteer computing, including a client, client GUI, application runtime system, server software, and software implementing a project web site.

Volunteer computing has been successfully used in high-energy physics, molecular biology, medicine, astrophysics, climate study, and other areas. Among them, we may highlight SETI@HOME that has sustained a processing rate of about 60 TeraFLOPS for several years [17]. Moreover, other volunteer computing projects have been developed in the field of bioinformatics such as POEM@HOME, FightMalaria@Home, Docking@Home, or GPUGrid.net (readers can visit https://boinc.berkeley.edu/projects.php).

This project is developed in the context of* Ibercivis* [18], which is an open framework created to deploy new volunteer computing platforms based on BOINC [15]. One of the challenges of Ibercivis is to allow the execution of several applications in the frame of a single BOINC project. We refer the reader to http://boinc.berkeley.edu/ for insights on how to run a volunteer project using BOINC.

### 2.3. Cost Estimation Model for Local GPU-Based Infrastructures

This section establishes the economic assumptions to assess the cost of our simulations in the local infrastructure. Equation (1) shows the cost of a given simulation in a local computer:

(1) $$\begin{array}{c} C_{{\text{local}}_{x}}=C_{e_{x}}+C_{m_{x}}+C_{c_{x}}, \end{array}$$

where *C*_local_*x*__ is the result of adding:

(i) *C*_*e*_*x*__: energy consumption costs:
  (2) $$\begin{array}{c} C_{e_{x}}=T_{{\text{local}}_{x}}\cdot e_{x}\cdot p_{e}\cdot m, \end{array}$$
  where *e*_*x*_ is the energy consumption for a given ligand *x* and *p*_*e*_ is the energy price. Both are expressed per unit of time.
(ii) *C*_*m*_*x*__: machine market price:
  (3) $$\begin{array}{c} C_{m_{x}}=\frac{p}{a_{t}}\cdot T_{{\text{local}}_{x}}\cdot m, \end{array}$$
  where *p* is the physical machine market price and *a*_*t*_ is the amortization per unit time. Typical values for the amortization period of a machine are 2-3 years. Note that *a*_*t*_ is based on the unit time; that is, if the unit time is minutes, then *a*_*t*_ = years · 365 · 24 · 60.
(iii) *C*_*c*_*x*__: machine collocation costs:
  (4) $$\begin{array}{c} C_{c_{x}}=\left(c_{t}\cdot m+A_{t}\cdot \left\lceil \frac{m}{m_{a}}\right\rceil \right)\cdot T_{{\text{local}}_{x}}, \end{array}$$
  where *c*_*t*_ is the collocation and *A*_*t*_ is the administrator salary, both of them are expressed in units of time. The adjustment is completed by specifying how many physical machines are assigned to an individual administrator (*m*_*a*_). The expression ⌈*x*⌉ corresponds to the ceiling function of *x*.

## 3. Experimental Setup

This section introduces the hardware-software environment for both the local and volunteer computing environments, the main features and input data sets of* BINDSURF*.

### 3.1. Hardware and Software Infrastructure

Our local experiments have been conducted in an Intel-based machine that is a high-performance platform composed of an Intel Xeon E5620 processor running at 2.4 GHz and an internal Nvidia Geforce 7300GT GPU. This card, which is tailored for graphics, is always connected to the system during the evaluation. Besides, five different Nvidia GPUs are connected separately to this system through the PCI express bus as accelerators; that is, only one of them is connected to the motherboard during the tests at a given time (see Table 1 for hardware specification). These cards are the following: the Nvidia GTX 465, which has enabled 11 SMs from the total of 16 in the GTX400 chip, the Nvidia GTX 480, which has 15 active SMs, the Nvidia Tesla C2070, which has 14 active SMs, and the Nvidia GTX 590, which is one graphics card made out of two graphics processors with up to 2 × 16 SMs. Additionally, we have also analyzed the last generation of Nvidia GPUs, that is, the Tesla K20c. CUDA toolkit 5.0 leverages Nvidia architectures. CPU-side is also targeted through GCC compiler 4.7.2 version and vectorization. The vectorization on Intel platforms is enabled by SSE extensions.

The Ibercivis project has many computational resources available to different scientific projects. Nowadays, the Ibercivis project offers up to 1597 nodes which include, at least, a GPU in the system. Those GPUs are classified into groups depending on their computing capabilities.  Table 2 shows different GPUs and operative systems available in those nodes. BINDSURF is based on Nvidia GPUs, so this feature limits the number of resources available for running our application. In our experiments, we use 16 machines out of 106 we have available. Finally, the BOINC server is configured to send each work up to three times. In case a work unit fails, the work is forwarded to another client; this guarantees fault tolerance in the overall system. Regarding the implementation of* BINDSURF* in the Ibercivis platform, we have chosen the GenWrapper option as the most convenient for allowing us to maintain the application within its original architecture. We have only made some minor changes in the source code to let the BOINC client know the percentage of work performed at a given point.

### 3.2. BINDSURF Parameters

We carried out VS calculations using* BINDSURF* for the prediction of representative ligand-protein cases. For our evaluations, three different ligand-protein cases are chosen, whose ligands conveniently represent chemical diversity of large compound databases. They are referred to as ligands A, B, and C. Ligand A is a blood clotting cofactor recently discovered by us [19]. Ligand B and ligand C have been extracted from their Protein Data Bank [20] complexes with the respective IDS 2*byr* and 3*p*4*w*. We have run 10 executions of* BINDSURF* per ligand for a given number of simulation steps.

Different Monte Carlo steps are taken into account, ranging from 5 to 50000, as an optimal value for this parameter does not exist for all different ligand types (A, B, and C). Therefore, it is convenient to perform VS calculations using different values of this parameter, since, sometimes, we might be interested in short simulations (steps = 5,10,50) for obtaining qualitative information about potential hotspots in the surface screening approach for millions of different ligands, but in some other situations we might be more interested in obtaining accurate predictions for a smaller set of ligands; thus we may use higher number of Monte Carlo steps such as 500,5000 and 50000. Lastly, it should be noted that, in general, it could be said that the running time is directly proportional to the number of Monte Carlo steps and to the type of ligand, as reported previously in the original BINDSURF publication [1].

## 4. Experimental Results

### 4.1. Performance Evaluation in the Local Infrastructure

Table 3 shows the execution times obtained in our local infrastructure. The execution times increase along with the number of simulation steps of Monte Carlo as expected. However, this increase is not linear since the execution time is dominated by input data preprocessing and other computations in BINDSURF.

Table 3 shows that the most time consuming simulation is when the simulation runs in Nvidia GPU GTX 465, whereas the simulation cost is reduced, as long as the experiments are executed in the most efficient platform as Tesla K20c.

### 4.2. Performance Evaluation in the Volunteer Computing Environment

Table 4 shows the execution time in seconds of BINDSURF for the execution of ligand A (l1c4), ligand B (2byr), and ligand C (3p4w) in the Ibercivis project. The execution time is divided intototal time andprocessing time. The latter is actually the percentage of computational resources used by our application in the client's computer. The former, however, includes the whole process of executing BINDSURF in the Ibercivis project, that is, including overheads previously described in Section 2.2.

The BINDSURF processing times in the computers offered by Ibercivis are equivalent to our lowest-line GPUs, which make sense as the major percentage of computers offered by Ibercivis clients are desktop machines which include only gamer-level cards. These GPUs offer great performance at a very low-cost price and, sometimes, even improve the performance results of the high performance line of Nvidia GPUs, code-named Tesla. This fact is shown for our smallest workloads, that is, below 500 Monte Carlo iterations. Table 4 also shows that the BINDURF total time is much higher than the processing time in Ibercivis. The total time involves several different tasks, including the processing time as previously explained in Section 2.2.

### 4.3. Cost Evaluation of Our Local Infrastructure

This section takes several economic assumptions to assess the cost of our simulations. They are as follows.

1. A machine from the local infrastructure costs $3,000 and the cost for the Nvidia GPUs is: $250 the GTX 465, $350 the GTX 480, $400 the GTX 590, $1300 the 12 Tesla C2070 and $3,000 the Tesla K20.
2. The amortization period of each of these machines is 3 years.
3. The KW/h price is that of Spain (http://www.statista.com/statistics/13020/electricity-prices-in-selected-countries/): $0.1352.
4. The collocation price per machine/year in the local infrastructure is $12,000.
5. The administrator salary is $3,300/month and each administrator is assigned to 100 machines from the local infrastructure.

The local infrastructure has two other additional costs due to the power consumption of our infrastructure and the economic costs that are included to buy and maintain such infrastructure. The real-time measurement of individual GPU components using a software approach is new and is only supported by the Nvidia GPU K20. This is done by using NVML (Nvidia management library) [21] and it reports the GPU's real-time power usage.

For the other cards, power dissipation measurements are obtained using the* Watts up?   .Net* power meter [22]. This device is connected between the power source and the power supply of the system and provides power dissipation information every second. Power information is logged by a different machine on the same room. Room temperature is controlled and set to 26°C during the measurements to minimize temperature impact on static power.

Table 5 shows the power consumption in our local infrastructure for the running times shown in Table 3. These values are the average for each set of experiments. The energy consumption in the local infrastructure increases along with the Monte Carlo simulation steps as expected.

Table 6 shows the economic costs of the BINDSURF simulation when processing 6000 redocking simulations of ligands of different types, that is, A, B, and C, and varying the number of Monte Carlo steps. These costs are based on (1), which have three main components as previously explained: energy consumption, machine market price, and collocation cost. The cost is averaged per unit of time.

## 5. Discussion

Volunteer computing is a distributed computing approach where citizens offer their own computing resources to solve scientific projects. Actually, this is the main advantage we find in this computational environment: the great computational power available for our project at no cost. Moreover, the ubiquity of GPUs and the ingenuity of the simulation community augur well for the scale and scope of future computational studies of biomolecules.

Table 6 shows the execution of BINDSURF costs in the range of 19K–33K$ for the largest simulation (50000 Monte Carlo steps) we have targeted in our experiments. The execution time for this simulation in our local infrastructure takes 2.5 hours on average (see Table 3). In Ibercivis, however, users have to wait up to 10.8 hours until the results of our simulation are returned back (see Table 4), although at no cost. It is also noteworthy that the cost of our simulations decreases along with the execution time and energy efficiency as expected (see Tables 3 and 6). As shown in Table 1, power consumption has been drastically improved in Tesla K20 and this is reflected in the overall simulation cost.

Several drawbacks are behind the use of volunteer computing as a platform to develop scientific computations. Firstly, it is a fully heterogeneous environment, making it difficult to leverage performance of the targeted architectures. However, the processing times obtained in the volunteer computing environment (see Table 4) are not that far from those taken in our local machines. At this stage of our research, we only focus on CUDA architectures that are relatively similar to each other. This would be a harder issue in an OpenCL-based machine as different kind of processors (i.e., CPUs, GPUs, and DSPs) might be targeted. Secondly, the success of the simulations depends on citizen's resources which are not always available. This is solved by having a large community of participants dedicated to a concrete project like we do in the Ibercivis project.

## 6. Conclusions and Future Work

Bioinformatics is an emerging research area which produces a great amount of HPC applications. Achieving the most performance in their execution is important but optimization through cost reduction is crucial. Researchers now have access to a seamless resource provision technology that is volunteer computing. Volunteer computing is a donation-based infrastructure applied to solving scientific projects and permits the user to forget about certain costs associated with physical infrastructures and also helps disseminate the project to the general public.

In this contribution we have evaluated the tuple BOINC and the Ibercivis Project for the drug discovery application* BINDSURF* running several relevant benchmarks. Focusing on the physical infrastructure, we have shown the execution times of our application for both the local and the Ibercivis infrastructure, finding that the processing times are in the same order of magnitude. We have provided with the information about power consumption measurements that our local infrastructure supports in order to run the* BINDSURF* application, and we have also provided an exhaustive cost model that considers a wide variety of elements and factors, allowing a detailed comparison with the execution of the same application on the infrastructure provided by Ibercivs. Besides, conclusions obtained from our study can be extrapolated to other GPU-based VS methodologies and bioinformatics applications.

However, volunteer computing is not the panacea, since it depends strongly on certain factors such as the concrete bioinformatics application, the size of the problem, and the compatible resources that are available for a project, and, thus, the optimal infrastructure may vary and it does not need to be always volunteer-based.

As future work, we plan to port* BINDSURF* to OpenCL [23], allowing its execution in a wider variety of heterogeneous computational systems such as multicore CPUs. This way, more work units types from Ibercivis participants could be harnessed, thus increasing the peak performance available for our application and also reaching broader public to disseminate our work. Moreover, a hybrid approach that mixes the use of public cloud providers, like Amazon, with the use of projects, such as Ibercivis, may improve the fault tolerance ratio of our simulations at low price.

## Figures and Tables

**Table 1:** Hardware features for our local test-bed infrastructure.

Intel system	
Processor	Intel Xeon E5620 @ 2.4 GHz
GPU 0	Nvidia 7300GT
Memory	16 GB DDR3 @ 1333 MHz
Maximum power draw	80 W
Experimental idle power	38 W

GPU 1: Nvidia GTX 465	
GPU family	GF100
Manufacturing process	40 nm
Core clock	607 MHz
Memory size	1024 MB
Memory clock	2 × 1603 MHz
Memory bus width	256 bits
Memory bandwidth	102.6 GB/sec
Stream processors	352
Maximum power draw	200 W
Experimental idle power	24 W

GPU 2: Nvidia GTX 480	
GPU family	GF100
Manufacturing process	40 nm.
Core clock	700 MHz
Memory size	1536 MB
Memory clock	2 × 1848 MHz
Memory bus width	384 bits
Memory bandwidth	177.4 GB/sec
Stream processors	480
Maximum power draw	250 W
Experimental idle power	37 W

GPU 3: Nvidia Tesla C2070	
GPU family	GF100
Process	40 nm.
Core clock	573.5 MHz
Memory size	6143 MB
Memory clock	2 × 1494 MHz
Memory bus width	384 bits
Memory bandwidth	143.4 GB/sec
Stream processors	448
Maximum power draw	247 W
Experimental idle power	107 W

GPU 4: Nvidia GTX 590	
GPU family	GF100
Manufacturing process	40 nm.
Core clock	1215 MHz
Memory size	2 × 1536 MB
Memory clock	2 × 1707 MHz
Memory bus width	2 × 384 bits
Memory bandwidth	2 × 327.7 GB/sec
Stream processors	1024
Maximum power draw	365 W
Experimental idle power	140 W

GPU 5: Nvidia Tesla K20c	
GPU family	GK110
Manufacturing process	28 nm.
Core clock	705 MHz
Memory size	5120 MB
Memory clock	2 × 2600 MHz
Memory bus width	320 bits
Memory bandwidth:	208 GB/sec
Stream processors	2496
Maximum power draw	225 W
Experimental idle power	27 W

**Table 2:** GPU-based machines in the Ibercivis project to date. The nodes are divided into operative systems and GPU brands.

	Windows	Linux	Darwin	Total
Nvidia	918	106	68	1092
ATI + Intel	445	10	50	505

Total	1363	116	118	**1597**

**Table 3:** Execution time in seconds of BINDSURF for the execution of ligand A (l1c4), ligand B (2byr), and ligand C (3p4w) in our local infrastructure with different Monte Carlo steps. Lowest RMSD values obtained for ligands B and C are 3 and 2 Angstroms, while there is no crystal structure available for ligand A.

Steps	GTX 465	GTX 480	GTX 590	Tesla C2070	Tesla K20
Ligand A (l1c4)					
5	57.79	57.70	59.11	58.57	68.52
10	57.97	57.97	57.64	59.15	68.78
50	61.66	61.73	62.08	63.68	72.40
500	114.17	114.09	118.37	126.62	116.66
5000	788.52	788.81	842.34	942.35	678.61
50000	8888.45	8890.91	9546.22	10702.00	7371.31

Ligand B (2byr)					
5	49.24	49.42	49.69	50.71	60.81
10	49.49	49.65	50.13	51.18	60.87
50	53.39	53.42	54.22	55.73	65.05
500	107.20	107.10	111.58	121.64	114.42
5000	733.35	732.86	783.29	891.92	686.69
50000	7162.95	7163.43	7688.34	8813.30	6569.21

Ligand C (3p4w)					
5	75.41	75.35	75.75	76.41	86.44
10	75.53	75.45	76.00	76.70	86.05
50	78.64	79.23	80.20	80.74	89.44
500	127.35	127.52	131.72	139.09	129.28
5000	761.68	761.46	813.74	903.31	640.37
50000	7925.02	7924.22	8520.64	9494.24	6219.92

**Table 4:** Execution time in seconds of BINDSURF for the execution of ligand A (l1c4), ligand B (2byr), and ligand C (3p4w) in Ibercivis with different Monte Carlo steps. It is divided into total time (i.e., including submission overheads) and processing time.

Steps	Total time	Processing time
Ligand A (l1c4)		
5	60.60	30893
10	61.88	24325
50	61.85	28469
500	130.79	31719
5000	1144.02	20840
50000	11467.09	28469

Ligand B (2byr)		
5	65.5	24158
10	64.58	24116
50	69.72	43664
500	133.73	91752
5000	1161.78	27727
50000	10895.33	42469

Ligand C (3p4w)		
5	72.82	46150
10	76.59	20016
50	86.77	1933
500	177.47	2016
5000	1190.07	31843
50000	12660.21	46150

**Table 5:** Averaged power consumption in Watts when processing ligand A (l1c4), ligand B (2byr), and ligand C (3p4w) in a local machine with different Monte Carlo steps. The runtimes are shown in Table 3.

Steps	GTX 465	GTX 480	GTX 590	Tesla C2070	Tesla K20
5	240.57	246.45	270.99	291.78	** 192.95**
10	236.79	246.50	271.44	293.32	** 193.72**
50	240.92	251.37	274.40	297.85	** 195.93**
500	276.09	293.75	309.07	325.52	** 214.83**
5000	312.79	351.90	355.19	357.10	** 245.57**
50000	318.79	362.36	364.24	356.14	** 253.95**

**Table 6:** Averaged economic costs in $ for processing 6000 redocking simulations of ligands (ligand A (l1c4), ligand B (2byr), and ligand C (3p4w)) in a local machine. We also vary the number of Monte Carlo steps to increase the computational cost.

Steps	GTX 465	GTX 480	GTX 590	Tesla C2070	Tesla K20
5	167.74	** 159.81**	162.16	166.99	200.20
10	166.28	** 160.33**	161.48	168.22	200.13
50	181.50	** 170.30**	172.72	180.08	210.55
500	394.53	** 306.64**	318.83	349.33	334.95
5000	3063.61	2017.71	2158.96	2475.37	** 1868.84**
50000	33020.51	21209.85	22812.22	26228.97	** 18797.83**
